# Notes on Pulp Technique

**Published:** 1903-06

**Authors:** Martha Anderson


### NOTES ON PULP TECHNIQUE.[1]

BY MARTHA ANDERSON, M.D.

THE comparative value of methods and stains used for nerve-tissue in other parts of the body have never (to my knowledge) been demonstrated on the pulps of the teeth. Our studies for the past four years has been along this line. While this work has not been complete, yet we are able to show some results. I herewith submit the results of my investigations of the past year.

In the work of preparing pulps for the demonstration of nerve-tissue the following methods of procedure have been carried out.

Fresh pulps have been hardened in a weak solution of formalin, in Müller's fluid, Erliki's fluid, and in Weigert's chrome alum solution, which formula is as follows:

---

[1] Abstract of a paper read before the American Medical Association, Section on Stomatology, New Orleans, La., May 5 to 8, 1903.

Chrome alum, two and one-half per cent.
Copper acetate (neutral), five per cent.
Acetic acid, five per cent.
Formalin, two to four per cent.
Dissolve the alum in the necessary water, add acetic acid, then copper acetate. Filter, and when cold add formalin.

Sections were cut by the freezing method, then stained by one of the following methods:

(*a*) Weigert's method for medullated nerves:

Copper acetate neutral sat. aq. sol. + aq. dest., āā (warm) 24 hours.
Wash.
Weigert's hæmatoxylin, 24 to 48 hours.
Weigert's differentiating sol., few minutes.
Wash.
Dehydrate clear and mount in balsam.

(*b*) Osmic acid and hæmatoxylin:

$OsO_4$, one per cent., ½ to 1 hour (dark).
Wash.
Pyrogallic acid, five per cent., 3 to 4 minutes.
Wash.
K. permanganate, one-fourth per cent., 3 to 4 minutes.
Wash.
Oxalic acid, one per cent., ½ minute.
Wash.
Counterstain in hæmatoxylin.
Wash, dehydrate, clear, and mount.

(*c*) Mummery's iron and tannin:

Wash sections in distilled water.
Liquor ferri perchlor. B. P., 24 hours.
Wash quickly in distilled water.
Tannic acid, gr. ii to ℥iss, 5 to 10 minutes.
Wash, dehydrate, clear, and mount.

(*d*) Iron and hæmatoxylin:

Zenker's fluid.
Wash.
Mordant, liquor ferri chlor., 24 hours.
Wash thoroughly.
Hæmatoxylin, one-half to one per cent. aq.
Wash well.
Decolorize weak mordant till pale straw.
Wash thoroughly (or will fade).
Dehydrate, clear, and mount.

(*e*) Freud's gold:

Wash water.
$AuCl_3$, one per cent., 3 to 5 hours (dark).
Wash.
Caustic soda, 1 to 5 or 6 water, 3 minutes dark.
Drain.
K. I., ten to twelve per cent., 5 to 15 minutes.
Wash, dehydrate, clear, and mount.

(*f*) Underwood's gold:

Wash in Na. bicarb. sol., gr. v to ℥i.
$SuCl_3$ neutralized by Na. bicarb. sol. gtt. by gtt., ½ to 1 hour dark.
Wash dist. water.
Formic acid, one per cent., warm and keep rather hot till crimson, ½ to 1 hour dark.
Dist. water, cold, ½ hour.
Dry.
Mount in glycerin jelly.
Avoid Canada balsam.

(*g*) Lemon-juice and gold:

Lemon-juice strained several times through flannel 10 to 15 minutes.
$AuCl_3$, one per cent. (dark), 1 hour.
Wash rapidly.
Formic acid, twenty-five per cent. (dark), 24 hours.
Transfer to glycerin and mount in glycerin.

Weigert's stain for medullated nerves was used on tissues hardened in Müller's fluid, formalin, and Weigert's chrome alum, and good results were obtained with both Müller's and formalin hardened tissues, the medullated sheaths of the nerves taking the stain.

The osmic acid method was used similarly to the above, and gave good results following Weigert's chrome alum solution.

Mummery's iron and tannin used after Müller's fluid stained the nerve-fibres, but the results were not so good as with the two previous stains. Mr. Howard Mummery, however, has been able by this stain to trace fine nerve-fibres from the nerve-bundles.

Iron and hæmatoxylin gave good results, the fibres in some places being traced to the apex of the pulp.

Of the different gold methods, Freud's has given me the best results, although some of the results of staining tissues hardened by all the methods have been good. Gold staining on the whole has been unsatisfactory. The staining has been unreliable. In carrying a number of sections through together by the same process, some will be acted upon by the stain, showing a good result; others will be unaffected.

With certain of the nerve-stains, the pulp-stones have taken the same stain as the nerve-tissues. The osmic acid method is an exception, the stain reacting differently upon the stones.

